# A Dominant Negative Zebrafish Ahr2 Partially Protects Developing Zebrafish from Dioxin Toxicity

**DOI:** 10.1371/journal.pone.0028020

**Published:** 2011-12-15

**Authors:** Kevin A. Lanham, Amy L. Prasch, Kasia M. Weina, Richard E. Peterson, Warren Heideman

**Affiliations:** 1 Department of Biomolecular Chemistry, University of Wisconsin, Madison, Wisconsin, United States of America; 2 School of Pharmacy, University of Wisconsin, Madison, Wisconsin, United States of America; 3 NimbleGen, Madison, Wisconsin, United States of America; 4 School of Pharmacy, University of London, London, England; Ecole Normale Supérieure de Lyon, France

## Abstract

The toxicity by 2,3,7,8 tetrachlorodibenzo-*p*-dioxin (TCDD) is thought to be caused by activation of the aryl hydrocarbon receptor (AHR). However, our understanding of how AHR activation by TCDD leads to toxic effects is poor. Ideally we would like to manipulate AHR activity in specific tissues and at specific times. One route to this is expressing dominant negative AHRs (dnAHRs). This work describes the construction and characterization of dominant negative forms of the zebrafish Ahr2 in which the C-terminal transactivation domain was either removed, or replaced with the inhibitory domain from the *Drosophila* engrailed repressor protein. One of these dnAhr2s was selected for expression from the ubiquitously active *e2f*α promoter in transgenic zebrafish. We found that these transgenic zebrafish expressing dnAhr2 had reduced TCDD induction of the Ahr2 target gene *cyp1a*, as measured by 7-ethoxyresorufin-O-deethylase activity. Furthermore, the cardiotoxicity produced by TCDD, pericardial edema, heart malformation, and reduced blood flow, were all mitigated in the zebrafish expressing the dnAhr2. These results provide *in vivo* proof-of-principle results demonstrating the effectiveness of dnAHRs in manipulating AHR activity *in vivo*, and demonstrating that this approach can be a means for blocking TCDD toxicity.

## Introduction

The aryl hydrocarbon receptor (AHR) is the ligand-activated subunit of a heterodimeric transcription factor found in numerous vertebrate cell types [1]. Agonist binding at the AHR PAS B domain triggers a conformational change that ultimately moves the normally cytosolic receptor into the nucleus where it dimerizes with its partner, the AHR nuclear translocator (ARNT). The activated heterodimer binds to canonical DNA sequences known as AHR Enhancers (AHREs) to drive transcription of target genes [2].

The AHR is activated by a variety of ligands, including some polycyclic aromatic hydrocarbons (PAHs), certain polychlorinated biphenyls (PCBs), and a number of other compounds. Activation of the AHR induces transcription of a core set of genes involved in xenobiotic metabolism. These include genes encoding cytochrome P450 1A (CYP1A), and UDP-glucuronosyltransferase (UDPGT) involved in biotransformation. Because these enzymes degrade the ligands that activate AHR, the system acts as an environmental sensor that facilitates the detoxification of xenobiotics [3]. The most potent AHR agonist known is 2,3,7,8 tetrachlorodibenzo-*p*-dioxin (TCDD). TCDD is noteworthy in being poorly degraded by the enzymes induced by AHR, and because it is lipophilic, this stable AHR agonist has a long half-life in biological systems.

Due to genome duplication events, zebrafish have multiple Ahr and Arnt isoforms, including three Ahrs (Ahr1a, Ahr1b, and Ahr2) [4], [5], [6] and two Arnts (Arnt1 and Arnt2) with multiple splice variants [6], [7]. Previous work has shown that Ahr2 and Arnt1c are responsible for TCDD toxicity in zebrafish; knocking down the expression of either of these proteins blocks most if not all toxicity [7], [8].

**Figure 1:**
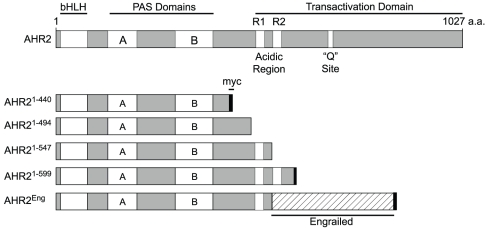
Maps of mutant AHRs. The full length Ahr2 is shown at top, with domains indicated. The different constructs made and tested are shown below.

The AHR protein can be divided into several domains (Fig. 1). An N-terminal basic helix-loop-helix domain is responsible for DNA binding. This is followed by two PAS domains, PAS A and PAS B. PAS domains are named for the Per-Arnt-Sim superfamily of proteins containing these domains, and are often associated with small ligands that influence conformation and signalling [9], [10], [11], [12]. These N-terminal domains are highly conserved between AHRs from different species. The C-terminal half of the AHR is responsible for mediating transactivation. In general, the C-terminus contains multiple transactivation domains (TADs) [13] including a bipartite acidic domain [14], a glutamine rich domain [13], [15], and a proline/serine/threonine rich domain [13]; however, the presence and function of these domains can vary among AHR orthologs [16], [17] and paralogs [5].

Although AHR activation can serve a protective role, it is known that inappropriate activation can induce a variety of toxic responses, especially in early development. This has led many to conclude that AHR also plays a role in controlling developmental processes [3]; however, the nature of this role remains obscure. Embryos are particularly sensitive to TCDD, and exposure during defined developmental windows disrupts normal morphogenesis [18]. Zebrafish embryos exposed to TCDD within the first few hours of fertilization exhibit multiple signs of toxicity. The most prominent signs of toxicity involve malformation of the skeleton, especially in the craniofacial region [19], and disruption of the cardiovascular system [20], [21]. Cardiovascular toxicity is characterized by reduced cardiac output, aberrant heart development resulting in a stretched string-like heart, pericardial and yolk sac edema, and reduced peripheral blood flow.

The fact that AHR activation by TCDD leads to tissue- and developmental-specific toxicity raises questions that can only be resolved by manipulation of AHR activity at specific times in specific tissues. The use of morpholino oligonucleotides (MOs) has enabled controlled knockdown of specific AHR and ARNT isoforms. However, MOs are limited in their ability to knock down gene expression in a temporally- and spatially-defined manner. More important, the MOs are generally injected into the newly fertilized egg and are only effective during the first few days after fertilization.

Work establishing the functions of specific AHR domains has made it possible to produce mutant AHR proteins with controlled function. For example, manipulation of the PAS-B domain can produce a constitutively active AHR [22].

It is also possible to diminish AHR function by removing key domains, specifically abolishing the TAD in the C-terminus. Dominant negative AHRs (dnAHRs) have been constructed by making C-terminal truncations of mouse and human AHRs, although their use has so far been limited to transient expression in cell culture assays [15], [23], [24]. A truncated human AHR, lacking the Q-rich and P/S/T domains, but retaining the acidic domain, has dnAHR activity [15]. Another dnAHR was engineered with a leucine to alanine mutation at position 678 that compromised the Q-rich TAD [15]. In the mouse, more severe truncations removing both the Q-rich, and the acidic TADs have been required to produce dnAHRs [24].

Expression of a dnAhr2 in zebrafish would allow manipulation of the AHR pathway *in vivo* without the use of agonist or antagonist chemicals. This would be effective beyond the narrow time limit of morpholino activity. In principle, tissue-specific expression of a dnAhr2 in zebrafish could be used to address key questions regarding the molecular mechanisms underlying TCDD toxicity.

Here we report the construction of a dnAhr2 and proof-of principle experiments using this to protect a transgenic zebrafish line from TCDD. We find that transgenic expression of a dnAhr2 produces highly significant, albeit incomplete, protection from several end points of TCDD toxicity.

## Methods

### Oligonucleotides

Oligonucleotides used for PCR were synthesized by Integrated DNA Technologies (Coralville, IA). All primers are written in the 5′ to 3′ direction. BK Reverse Primer: ACA GGA AAC AGC TAT GAC CTT G. Ahr2^1–440^mycB rev: CTA TCG CGA CAA GTC CTC TTC AGA AAT AAG CTT TTG CTC AGG ACT CGA GAT TTT CTG GCC TTT GCT CTG. Ahr2^1–494^ rev: CTA CTC GAG GAC GGC TGC CAG GTG TTC CC. Ahr2^1–547^ rev, CTA CTC GAG CTG CAC AGA GTG TTC TCC CA. Ahr2^1–599^mycB rev: CTA TCG CGA CAA GTC CTC TTC AGA AAT AAG CTT TTG CTC ATT CCT CGA GTC CAA CTC TGA TAG ACC CTC. EngFXhoI: ATA CCG TCT ACC TCG AGA GCC. EngRApaI: GGG CCC AAG CTT GAT CCC AGA GCA GAT. mycEngR2 rev: GGG CCC CTA TCG CGA CAA GTC CTC TTC AGA AAT GAG CTT TTG CTC GAA GCT TGA TCC CAG AGC AGA. BglIIF: AGA TCT CCC GGG CTG CAG GAA TTC GAT. BglIIR: AGA TCT CTA TCG CGA CAA GTC CTC TTC.

### Plasmid Construction

#### Truncated pBKCMV-Ahr2 Plasmids

The truncated forms of Ahr2 were amplified with PfuTurbo polymerase (Stratagene, La Jolla, CA) using the previously described pBKCMV-Ahr2 plasmid [25]. The BK reverse primer was used as a forward primer, this amplified a portion of the native 5′ UTR with a BamHI site on the 5′ end. Reverse primers are as indicated for each truncation product. The primers for Ahr2^1–440^ and Ahr2^1–599^ added a 1× myc tag to the amplicon. Amplified products for Ahr2^1–440^, Ahr2^1–494^, Ahr2^1–547^ and Ahr2^1–599^ were TA sub-cloned into the pGEM-T Easy vector (Promega) and sequenced. Constructs were digested out of pGEM with BamHI and NotI and inserted into pBKCMV at these sites.

#### pBKCMV-Ahr2^Eng^

For the construction of pBKCMV-Ahr2^Eng^ the repressor domain of Engrailed was Pfu amplified from the pks/En vector template using EngFXhoI and EngRApaI primers. The amplified product was TA sub-cloned into pGEM-T Easy vector and sequenced to make pGem Engrailed. pGem Engrailed was digested with XhoI and ApaI and inserted into pBKCMV-Ahr2 at these sites to make pBKCMV-Ahr2-Engrailed. A myc tag was added to this construct by Pfu amplifying the Ahr2-Engrailed fusion with the BK reverse primer and mycEngR2 rev. The resulting product was TA sub-cloned into pGEM-T Easy and sequenced. The insert was then digested out with BamHI and NotI and placed back into a pBKCMV vector to make the final myc tagged pBKCMV-Ahr2^Eng^ plasmid.

#### Ef1-α Ahr2^1–440^

The Ahr2^1–440^ sequence was Pfu amplified with the BglIIF and BglIIR primers using the pBKCMV-Ahr2^1–440^ vector as a template, which flanked the insert with BglII sites. The amplified product was sub-cloned into pGEM-T Easy and sequenced. The Ahr2^1–440^ sequence was excised from pGEM-T Easy using BglII and inserted into the pW1x/B Ef1-α construct (a generous gift from Elwood Linney) at BglII. Orientation was determined by restriction digest.

#### Ef1-α Ahr2^Eng^

The Ahr2^Eng^ sequence was Pfu amplified with the BglIIF and BglIIR primers using the pBKCMV-Ahr2^Eng^ vector as a template, which flanked the insert with BglII sites. The amplified product was sub-cloned into pGEM-T Easy and sequenced. The Ahr2^Eng^ sequence was excised from pGEM-T Easy using BglII and inserted into the pW1x/B Ef1-α construct at BglII. Orientation was determined by restriction digest.

### Transient Transactivation Assay

COS-7 cells were seeded on 24-well plates at a density of 5×10^4^ cells/well 1 day before transfection [5], [26]. Transfections were performed using SuperFect (QIAGEN). Each well was co-transfected with 400 µl of serum-containing media containing pBKCMV expression constructs for Ahr2 (225 ng) and Arnt2b (450 ng). For dominant negative testing, Ahr2^1–440^, Ahr2^1–499^, Ahr2^1–547^, Ahr2^1–599^ or Ahr2^Eng^ (225 ng each) were added to the assay as indicated. When a dnAHR plasmid was not added, empty vector was included so that in all assays the total amount of plasmid - both expression plasmid and reporters remained constant.

The pGudluc 1.1 reporter vector was obtained from Dr. Michael Denison (University of California, Davis, CA). This reporter vector is based on pGL2-Basic and has the firefly luciferase gene under control of a 484-bp fragment of the mouse CYP1A1 enhancer that contains 4 DREs and the murine mammary tumor virus promoter [27]. The *pGudluc* luciferase reporter construct (100 ng) was included in all transfections as indicated, along with a β-galactosidase CMV reporter (100 ng) to control for transfection efficiency.

Media was removed by vacuum aspiration, each well was washed with 1×PBS and 100 µl of passive lysis buffer was added. Plates were incubated 20 min at room temperature on an orbital shaker. Cell lysis was confirmed microscopically and 10-µl aliquots were transferred to a 96-well luminometer plate. Luminescence assays were completed using a Dynatech Laboratories ML-2250 luminometer (Chantilly, VA) as follows: 50 µl of luciferase assay buffer II was injected into each well, incubated 2 s, and the resulting luminescence integrated over the next 10 s.

Luciferase activity was normalized to β-galactosidase activity for each sample [26]. Vector controls with no AHR or ARNT expression produced no luciferase activity. Significance was determined using a two-tailed Student's t-test assuming unequal variances.

### Microinjection of Ahr2 Constructs

Fertilized eggs were obtained from adult AB strain zebrafish bred in our laboratory as described by Westerfield (1995). Newly fertilized eggs were injected with either Ahr2^1–440^ or Ahr2^Eng^ at the one- to two-cell stage with approximately 13 ng of linearized plasmid. Zebrafish were grown to maturity at 27°, outcrossed to wild type AB fish and the resulting embryos were collected for PCR analysis to determine whether they produced transgenic offspring [28].

All work was done in an ALAC-accredited facility with an IACUC-approved animal care and use protocol # M489 approved by the University of Wisconsin School of Medicine and Public Health (SMPH) Animal Care and Use Committee. Moreover, much of the motivation for the work is based on an interest in animals and this guided efforts to ameliorate any suffering. In fact, all experimental work utilized embryos at very early stages of development. While there is no way a human can know how an animal feels, our best estimates indicate that suffering is connected with complexity in behavioral repertoire. These small embryos show few signs of distress and were often terminally anesthetized in our experiments.

### Waterborne TCDD Exposure of Zebrafish Embryos

TCDD of >99% purity obtained from Chemsyn (Lenexa, KS) was dissolved in dimethyl sulfoxide and used to make a 1 µg/mL TCDD stock solution. Embryos (2–4 hours post-fertilization) were statically exposed to vehicle (0.1% dimethyl sulfoxide) or TCDD (1.0 ng/ml) for 1-h in glass scintillation vials with gentle rocking as described [29]. After the 1-h static exposure, embryos were rinsed with clean water and maintained in vehicle/TCDD-free water for the remainder of experiments.

### Ethoxyresorufin O-deethylase Assay

An *in vivo* EROD assay was used to assess Cyp1A activity [30]. Embryos were exposed to 7-ethoxyresorufin (0.4 µg/ml, Sigma) for 5 min and immobilized in 3% methylcellulose for epifluorescence microscopy (excitation λ, 577 nm; emission λ, 620 nm).

### Western Blot

Embryos (20/sample, 72 hpf) obtained from transgenic lines or from a wild type AB strain were euthanized with MS-222 (1.67 mg/ml, Sigma-Aldrich, St. Louis, MO), homogenized with a microfuge tube pestle in 40 µL of 2× SDS sample buffer, heated at 95° for 5 min, centrifuged at 13,000 g for 10 min, and the supernatants were loaded onto an 8% SDS-polyacrylamide gel. As a positive control, Ahr2^1–440^ protein was translated *in vitro* using the TNT T7/T3 Coupled System (L5040, Promega) and the myc tagged pBKCMV-Ahr2^1–440^ plasmid as the template. Proteins were transferred to a nitrocellulose membrane, and probed using an anti-myc antibody (MS-139-P0, Lab Vision Corporation, Fremont, CA). Protein was visualized using the ECL Plus detection kit (Amersham) followed by exposure to radiographic film.

### Toxicity Assessment

Embryos were obtained from Line 26 or the isogenic AB wild type spawns just after fertilization. Exposures consisted of 20 embryos from each group placed in water and statically exposed to TCDD (1 ng/ml, 2 ml) or vehicle for 1 h, rinsed with clean water, and sorted into 96-well plates (1 embryo/well) [20], [29]. Each toxicity experiment was repeated 4 times. At 96 hpf the larva were scored for severity of pericardial edema, defects in heart looping, and reduced caudal blood flow. Pericardial edema was assessed from a lateral view; heart looping was assessed from a ventral viewpoint. For blood flow assessment larva were observed laterally and the rate of flow as blood traversed from the caudal artery to the caudal vein was scored. The scale used was: 0 - no defect; 1 - defect difficult to detect; 2 - defect clearly present; 3 - defect obvious and severe. Results are the average of four replicates from two independent experiments. Significance was determined using a two tailed Student's t-test assuming unequal variances. Assessments were performed using a Nikon Eclipse TE300 inverted microscope.

## Results

### Design and Construction of dnAhr2s

We hypothesized that loss of the TAD would produce an AHR that could interact with ARNT and AHREs without yielding transactivation, potentially competing at the protein and DNA binding sites with endogenous AHR to produce a dominant negative effect. The TAD is poorly conserved across species, and work with mouse and human AHRs has demonstrated that changes producing a dominant negative receptor in one AHR isoform do not necessarily produce a dnAHR in another isoform. As shown in Figure 1, our approach was to make progressive deletions of the Ahr2 C-terminus. The smallest deletion removes the Q-rich site, but keeps the acidic domain intact. Further deletions bisect, and entirely remove the acidic domain. As an alternative strategy, we attached the repressor domain from the *Drosophila melanogaster* Engrailed protein to Ahr2^1–541^ in an attempt to actively repress transcription at Ahr2 target genes. Some of the constructs were tagged with a myc epitope, allowing western blotting with a commercially available monoclonal antibody.

### Ahr2^1–440^ and Ahr2^Eng^ Act as dnAhr2s in Cell Culture

**Figure 2:**
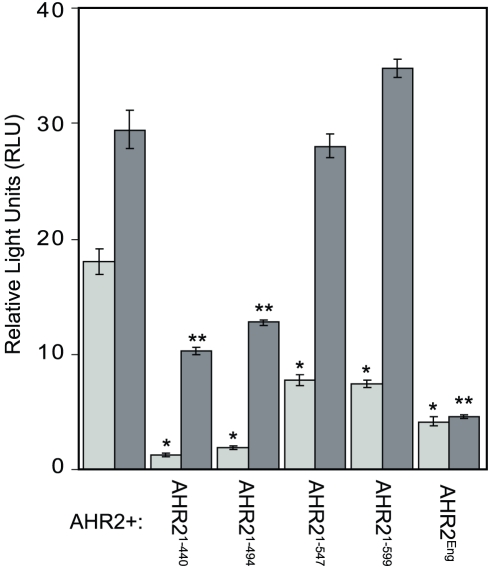
*In vitro* transcription assays show dominant negative activity. A Cos-7 cell transfection assay described in the Materials and Methods was used to measure the ability of Ahr2 and ARNT to induce luciferase reporter expression when different Ahr2 mutants were co-expressed in the system. Light bars indicate activity in the absence of TCDD; dark bars indicate activity in the presence of TCDD (1 ng/ml). Units are Relative Luciferase Units, a dimensionless ratio of luciferase activity from the luminometer divided by the β-galactosidase activity from the control reporter used for transformation normalization [5], [26]. Single asterisks: different from control no TCDD value, p<0.05. Double asterisk significantly different from control +TCDD value, p<0.05.

To test the mutant Ahr2s as dominant negatives, each construct was co-transfected into a Cos7 cell assay system together with expression plasmids encoding wild type Ahr2, and Arnt2b as well as the pGudLuc1 luciferase reporter plasmid [27], [31]. By measuring luciferase activity in the presence of vehicle, or 10 nM TCDD, we could determine how each mutant Ahr2 affected normal Ahr2/Arnt2b transactivation (Fig. 2). As previously reported, the system produced luciferase activity even in the DMSO control; however, TCDD further increased luciferase activity [8], [31].

The Ahr2^1–547^ and Ahr2^1–599^ constructs, retaining at least part of the acidic domain, did not reduce reporter activity with TCDD present, but did reduce the basal reporter activity in the absence of TCDD. In contrast, Ahr2^1–440^, Ahr2^1–494^, and Ahr2^Eng^ inhibited both constitutive luciferase activation, and activation following TCDD exposure. Addition of the Engrailed domain converted Ahr2^1–547^, which did not antagonize Ahr2 activation on its own, into a dnAhr2.

### Construction and Validation of dnAhr2 Transgenics

**Figure 3:**
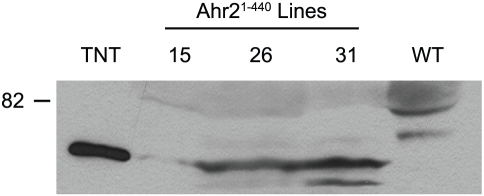
Western blot confirms expression of Ahr2^1–440^ in transgenic zebrafish lines. Embryos from the transgenic lines 15, 26 and 31, carrying the Ahr2^1–440^ transgene were processed for western blotting with an anti-myc antibody as described in Materials and Methods. The lane carrying the mutant Ahr2^1–440^ protein made *in vitro* for use as a positive control is indicated as TNT, and a negative control lane loaded with extract from wild type embryos having no myc tag is indicated as WT.

We chose Ahr2^1–440^, and Ahr2^Eng^, the two strongest dnAhr2s, to make transgenic zebrafish lines expressing the dnAhr2s from the *Xenopus laevis* EF1-alpha promoter. PCR screening identified putative germ-line positive founders, and these were further tested using western blotting to confirm the presence of the epitope-tagged protein in F1 embryos. Several putative Ahr2^Eng^ lines were identified by PCR, but unfortunately none passed our western blot screen for Ahr2^Eng^ protein expression. However, we found three independent lines (15, 26 and 31) that express the Ahr2^1–440^ transgene (Fig. 3). These produced a protein of the expected molecular weight, matching the mobility of the protein expressed in a cell-free lysate (TNT), and the band was not seen at all in control extracts made from untagged wild type (WT) embryos. We chose line 26 for further study.

### Expression of Ahr2^1–440^ Inhibits EROD Induction by TCDD

**Figure 4:**
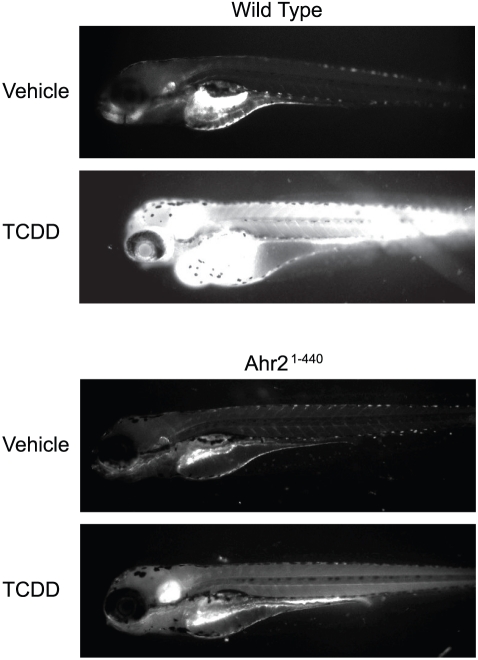
Induction of Cyp1a by TCDD is inhibited in dnAhr2^1–440^-transgenic zebrafish. Transgenic and wild type AB fish were exposed to TCDD or vehicle and ethoxysresorufin was added as described in Materials and Methods. Representative images showing the fluorescent product of Cyp1a metabolism are shown for wild type AB and dnAhr2^1–440^ transgenic fish.

Activation of AHR by TCDD produces a massive induction of cytochrome P4501a (Cyp1a) mRNA [32]. The Cyp1a oxidase converts 7-ethoxyresorufin into the fluorescent product resorufin. This allows AHR activity to be monitored *in vivo* by using fluorescence microscopy [30] (Fig. 4). All zebrafish produce autofluorescence in some structures, especially at the yolk as shown in the control images; however, this is very slight when compared to the intense fluorescence produced in wild type embryos exposed to TCDD. This intense fluorescence is not observed in the absence of the substrate (not shown). The TCDD-exposed embryos also displayed hallmark signs of TCDD toxicity including pericardial and yolk sac edema. In marked contrast, the transgenic zebrafish expressing Ahr2^1–440^ showed very little fluorescence in the presence of TCDD. Exposure to vehicle alone produced no fluorescence aside from autofluorescence normally seen in the yolk and around the margins of the fish [32]. Thus, we detected no evidence of Cyp1a in the absence of TCDD, while in the presence of TCDD EROD activity was consistently and clearly reduced by the presence of the Ahr2^1–440^ transgene.

### *In Vivo* Expression of Ahr2^1–440^ Protects Against Pericardial Edema

**Figure 5:**
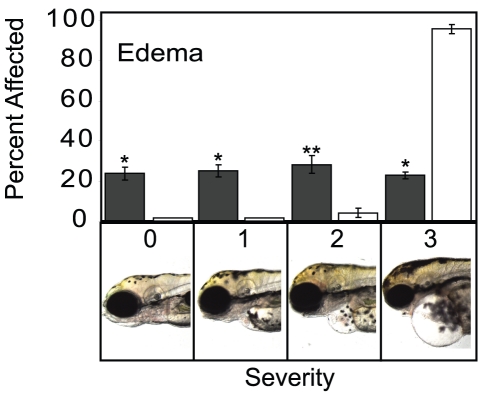
dnAhr2^1–440^ -transgenic zebrafish are resistant to pericardial edema caused by TCDD. Transgenic and wild type AB zebrafish were exposed to TCDD immediately following fertilization, and the severity of pericardial edema was scored for each fish at 96 hpf. For each type of fish, the bars indicate the distribution across each of the 4 possible categories of severity: Dark bars indicate dnAhr2^1–440^-transgenics; light bars indicate wild type. Representative photographs illustrate each severity score. Asterisk indicates a significant difference between the transgenic and wild type percentage for that severity category, p<0.05.

Pericardial and yolk sac edema are hallmark signs of TCDD toxicity in early life stage fish. This edema formation is probably secondary to heart failure induced by TCDD [20]. If the Ahr2^1–440^ truly acts as a dnAHR, then it should protect the zebrafish embryos from pericardial edema produced by TCDD. To test this, wild type and dnAhr2^1–440^ transgenic zebrafish embryos were exposed to TCDD (1 ng/ml) for 1 hour immediately following fertilization. Nearly 100% of the wild type embryos developed pericardial edema by 72 hpf, and this became severe by 96 hpf. In contrast, several of the offspring derived from the Ahr2^1–440^ cross showed no pericardial edema at all, and approximately 75% showed at least partial protection (Fig. 5).

### Ahr2^1–440^ Protects against heart looping defects caused by TCDD

**Figure 6:**
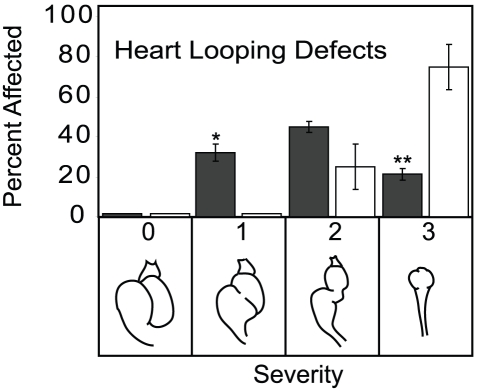
TCDD effects on heart morphology are reduced in dnAhr2^1–440^-transgenic zebrafish. Transgenic and wild type AB zebrafish were exposed to TCDD immediately following fertilization, and the severity of defects in heart looping and morphology were scored for each fish at 96 hpf. For each type of fish, the bars indicate the distribution across each of the 4 possible categories of severity: Dark bars indicate dnAhr2^1–440^-transgenics; light bars indicate wild type. Representative sketches of heart morphology illustrate each severity score. Asterisk indicates a significant difference between the transgenic and wild type percentage for that severity category, p<0.05.

Wild type zebrafish embryos exposed to TCDD initially form a normal heart, with the atrium looping dorsally and to the left so that it sits above the ventricle. However, by 72 hpf the hearts of TCDD-exposed embryos begin to unloop, eventually forming a linear, tube-like heart, such that the atrium sits behind, and in line with the ventricle. In an experiment similar to that described above, wild type and Ahr2^1–440^ transgenic zebrafish were exposed to TCDD and examined at 96 hpf. Nearly 80% of wild type offspring exposed to TCDD displayed the expected severe defect in heart looping, with an elongated, tube-like heart. In contrast, almost 80% of dominant negative offspring were scored as having only mild or moderate defects in heart looping (Fig. 6). Approximately one quarter of the dnAhr2^1–440^ offspring had hearts that were still looped to the extent that the chambers overlapped, lying adjacent to each other. This was never observed in wild type embryos exposed to TCDD. These results show that the dnAhr2^1–440^ can reduce the cardiotoxicity of TCDD in developing zebrafish.

### Expression of dnAhr2^1–440^ protects against reduced blood flow

**Figure 7:**
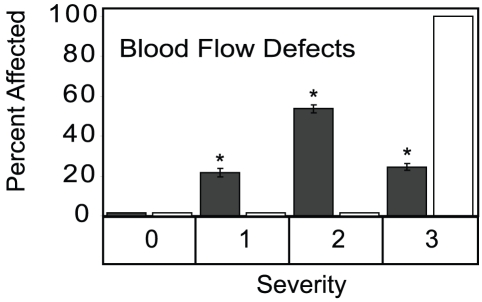
TCDD-induced blood flow defects are reduced in dnAhr2^1–440^-transgenic zebrafish. Transgenic and wild type AB zebrafish were exposed to TCDD immediately following fertilization, and at 96 hpf caudal blood flow was scored for each fish. Scores were: 3, no flow; 2, clearly diminished flow; 1 slightly diminished flow; 0 normal flow. The bars indicate the distribution across each of the severity categories: Dark bars indicate dnAhr2^1–440^-transgenics; light bars indicate wild type. Representative photographs illustrate each edema severity score. Asterisk indicates a significant difference between the transgenic and wild type percentage for that severity category, p<0.05.

Reduced caudal blood flow is evident by 72 hpf in TCDD exposed embryos [29], [33]. By 96 hpf, caudal blood flow was severely reduced or absent in 100% of wild type embryos exposed to TCDD (Fig. 7). However, the dnAhr2^1–440^ offspring were protected against reduction in caudal blood flow, comparable to the protection conferred against the heart looping defects. While TCDD treatment halted blood flow in the wild type embryos, blood flow continued in those embryos carrying the dnAhr2^1–440^ transgene.

## Discussion

### AHR2 Structure Activity

Our *in vitro* assays for dnAhr2 activity allowed several conclusions to be drawn regarding the zebrafish Ahr2. The most obvious conclusion was that we needed to delete almost the entire TAD in order to see a dominant negative effect in competition assays with full-length Ahr2.

Because the Ahr2^1–547^ and Ahr2^1–599^ mutants had no dnAHR activity, we initially concluded that they retained some normal function. However, in direct transactivation assays comparing the activities of different Ahr2 mutants cotransfected with ARNT, the Ahr2^1–599^ had only a small level of activity, despite retaining both acidic domains (Fig. S1). The acidic domain of the zebrafish Ahr2 is therefore functional as a transactivation domain, but only weakly so alone. Some other portion of the C-terminus is responsible for most of the transactivation potential of Ahr2.

The Ahr2^1–547^ and Ahr2^1–599^ constructs did not inhibit TCDD activated Ahr2; however, they did reduce basal activity. We can only speculate about this since the basis for the commonly observed high basal activity in the transfection assay has never been identified. It is possible that in Cos7 cells, Ahr is expressed at high enough levels to enter the nucleus unliganded and complex with ARNT by mass action. Another possibility is that unidentified AHR agonists are present in the cell culture system. Both models suppose the presence of low levels of AHR/ARNT in the nucleus without TCDD. The Ahr2^1–547^ and Ahr2^1–599^ constructs might interfere with transactivation caused by low basal levels of Ahr2 in the nucleus, but not be able to inhibit the higher Ahr2 levels produced by TCDD.

In contrast to the results obtained with Ahr2^1–547^ and Ahr2^1–599^, the co-expression of Ahr2^1–440^, Ahr2^1–494^, and Ahr2^Eng^ with Ahr2 inhibited both constitutive luciferase activation, and activation following TCDD exposure. Addition of the Engrailed domain strongly repressed luciferase induction and converted Ahr2^1–547^, which cannot antagonize Ahr2 activation on its own, into a strong dominant negative receptor.

### Mechanism of dnAhr2 Action

The COS7 cells were used because they have little if any detectable endogenous AHR or ARNT activity. This makes a good platform for adding different AHR and ARNT isoforms and testing them with a luciferase reporter. While qualitatively useful, the assay consistently has an unusually high basal response with no TCDD added; TCDD addition then increases activity, but the induction is only on the order of 2 fold. This is not an accurate representation of the normal induction of the CYP1a1 promoter by TCDD-activated AHR, in which basal activity is low, and fold induction is high. The high COS-7 basal activity requires expression of both AHR and ARNT [5], [6], [7], [8], [25], [26], [31], [34].

The Ahr2^1–440^ construct had a greater impact on basal than TCDD-induced activity. Both were reduced; however if one assumes that the TCDD-induced activity represents the sum of the basal and added TCDD-induced activity, then it is possible that the Ahr2^1–440^ effect is due solely to a block of basal activity.

An alternative interpretation is that Ahr2^1–440^ blocks both the basal and the induced activity, but blocks the basal activity more effectively; perhaps because the basal activity is, for reasons unclear, weaker.

Although the COS-7 cell transfection assay has been widely used, the source of the high basal activity remains obscure, and the *in vitro* results do not distinguish between these possibilities. However, the *in vivo* results clearly show reduced TCDD-induced Cyp1a activity. This EROD activity is known to be dependent on both AHR and Cyp1a. Furthermore, in the zebrafish embryo, basal EROD activity is undetectable in our assay. Therefore, subtraction of basal activity could not account for the reduction in TCDD-induced EROD activity observed in the strain carrying the Ahr2^1–440^ transgene. The Ahr2^1–440^ transgene must have impacted TCDD-induced AHR activity.

It is likely that further efforts to obtain the Ahr2^Eng^ transgenic line would yield a strain with a stronger dnAHR effect; however, our goal was to demonstrate that expression of an altered mutant AHR could be used to block TCDD effects in a living organism. The Ahr2^1–440^ served this purpose.

A dominant negative could act by sequestering ARNT in a complex unable to bind to AHREs, leaving no remaining ARNT protein molecules for productive interactions with Ahr2. The dnAhr2 might form a complex with ARNT that can bind AHREs but cannot regulate transcription. Binding to ARNT is required for both mechanisms. Deletion of the entire C-terminal half was needed to produce dnAhr2 activity. This suggests that dominant negative activity stems from the production of an intact DNA binding and dimerization module, without any remaining TADs. This idea is strengthened by our ability to create a dnAHR by adding the engrailed repressor to the C-terminus.

### dnAhr2 and TCDD Toxicity

The Ahr2^1–440^ transgenic fish did not display any developmental abnormalities, which is consistent with previous reports where Ahr2 was knocked down in zebrafish using morpholinos [8]. The construction of *Ahr* (−/−) null mouse lines has indicated a developmental role for the mouse AHR in a number of tissues [35], [36], [37], [38], [39]. However, our results suggest that inhibition of endogenous Ahr2 in our transgenic line was not total and therefore we cannot address whether zebrafish Ahr2 has a role during normal development, as low levels of active Ahr2 protein may be sufficient to perform potential developmental functions. Regardless, we observed no effects of the transgene on untreated animals and they grew and developed normally.

Fish from all 4 groups - line 26 and wild type; treated and untreated - survived until the 96 hpf point to the same extent. This is because the transgene by itself did not affect survival, and TCDD-induced lethality does not occur until after 120 hpf. Therefore, very similar numbers of individuals were scored in all 4 groups. Of the groups of 20 embryos chosen for each assay, survival at 96 hpf ranged from 15–20, with no individual treatment or strain consistently showing greater survival over the 4 independent experiments.

At 96 hpf the Ahr2^1–440^ transgenic fish showed very obvious protection against TCDD toxicity. However, the protection was rarely complete. Furthermore, we were not able to bring any of the Ahr2^1–440^ transgenic fish to adulthood after TCDD exposure, even though their appearance showed marked protection. This may be due to the summation of partial toxicities. One point to bear in mind is that the exposure to TCDD was at 1 ng/ml, while exposure of the same duration at 100 pg/ml is sufficient to produce 100% mortality. Survival to adulthood requires the completion of numerous hurdles, and our dominant negative protein may simply not be able to protect against all actions of TCDD, nor completely block AHR hyperactivation.

The effects of the transgene were remarkably specific, being observable only when the receptor target for the dnAHR was activated by TCDD. We have used a variety of transgenic zebrafish carrying both transgenic reporters and functional proteins with this *ef1-α* plasmid and other vector backbones. None of these have protected against TCDD toxicity. Therefore, the effect cannot be ascribed to the vector.

The basis for dominant negative proteins first proposed by Herskowitz [40] is that competition will occur between the normal protein and the mutant protein for some key cellular component. This competition mechanism ties the degree of effect of the dominant negative to its level of expression, relative to the level of the normal protein. With this in mind, the partial rescue of TCDD toxicities is not surprising at all.

To be effective as a competitor, the dnAhr2 must be expressed at levels sufficient to overwhelm the endogenous AHR2. It is not clear whether the EF1-alpha promoter produces expression levels that are sufficient to accomplish this in all tissues *in vivo*. Tissue specific silencing of EF1-alpha has been reported [41].

Despite these shortcomings, our proof-of-principle experiments clearly show the potential for manipulating the AHR pathway using dnAHRs in transgenic animals. This opens the doorway to a variety of whole animal experiments in which inducible, tissue-specific promoters can be used to downregulate AHR signaling in specific tissues at specific times. This extends our ability to study AHR function, TCDD toxicity, and development in a whole animal vertebrate system.

## Supporting Information

Figure S1 ***In vitro*** **transactivation assays show Ahr2^1–599^ activity.** The Cos-7 cell transactivation assay similar to that described in Figure 2 was used to measure the transcriptional activity of different Ahr2 mutants when paired with Arnt2b. Light bars indicate activity in the absence of TCDD; dark bars indicate activity in the presence of TCDD (1 ng/ml). Asterisks indicate activity that is significantly higher than the control cells expressing no Ahr or ARNT, p<0.05.
(EPS)
